# Low neighbor of Brca1 gene expression predicts poor clinical outcome and resistance of sunitinib in clear cell renal cell carcinoma

**DOI:** 10.18632/oncotarget.21999

**Published:** 2017-10-23

**Authors:** Wen Xiao, Zhiyong Xiong, Changfei Yuan, Lin Bao, Di Liu, Xiong Yang, Wencheng Li, Junwei Tong, Yan Qu, Lei Liu, Haibing Xiao, Hongmei Yang, Xiaoping Zhang, Ke Chen

**Affiliations:** ^1^ Department of Urology, Union Hospital, Tongji Medical College, Huazhong University of Science and Technology, Wuhan 430022, China; ^2^ Department of Pathogenic Biology, School of Basic Medicine, Huazhong University of Science and Technology, Wuhan 430030, China

**Keywords:** NBR1, clear cell renal cell carcinoma, prognostic markers, chemoresistance

## Abstract

**Objective:**

To study the expression of Neighbor of Brca1 gene (NBR1) in clear cell renal cell carcinoma (ccRCC), renal cancer cells and the chemoresistance cells and to elucidate its clinical prognostic and chemoresistance value.

**Materials and Methods:**

We screened the NBR1 mRNA in ccRCC from The Cancer Genome Atlas (TCGA) database and examined expression levels of NBR1 mRNA in 48 cases of ccRCC tissues, renal cancer cell lines and chemoresistance cells by qRT-PCR. Then, we extended two additional data sets in oncomine datebase (https://www.oncomine.org) to further confirm the results of the TCGA database. Immunohistochemistry (IHC) assay data performed in ccRCC tissues and normal tissues were downloaded from The Human Protein Atlas.

**Results:**

The mRNA levels of NBR1 were downregulated in TCGA-KIRC database (n = 533) and ccRCC patient samples (n=48) as well as in RCC cell lines and their chemoresistance cells. Similarly, the protein levels of NBR1 were lower in ccRCC patient samples. NBR1 level was associated with the clinical pathological stage and could discriminate metastasis, recurrence and prognosis in ccRCC patients. Low level of NBR1 mRNA showed a significance poor prognostic of overall survival (OS), disease–free survival (DFS) with univariate and multivariate analyses in ccRCC patients and sunitinib resistance.

**Conclusions:**

Taken together, our results suggest that low level of NBR1 can predict poor clinical outcome and resistance of sunitinib in patients with ccRCC.

## INTRODUCTION

Renal cell carcinoma (RCC), which constitutes 3.79% of all adult malignancies, is the most common and lethal urological malignancy in United States [1]. Approximately 70% to 80% of all RCC histological subtype is clear cell renal cell carcinoma (ccRCC) which has the highest rate of mortality, invasion, metastasis and chemoresistance [2]. Recent a study showed 90.7% patients of three major RCC histological subtypes had been ccRCC in their analysis [3]. Patients with ccRCC were normally treated with standard surgical resection but varied greatly in the outcome, as 51% patients had died with a median of 1.9 years after surgery, 4%-45 % of patients with locally limited tumors and 64% to 88% of them with advanced tumors passed away after ten years of nephrectomy [4]. Metastasis and invasion leads to 90 % of cancer-related deaths and a poor outcome of all cancers including ccRCC [5]. Recent pharmaceutic outcomes in the VHL-HIF2α-angiogenesis pathway of ccRCC which based on specific molecular target drugs such as the inhibitor of receptor tyrosine kinases (RTKs) had changed the treatment landscape for patients with metastatic ccRCC [6, 7]. Sunitinib had been used as first-line therapy of advanced ccRCC as it was a broad-spectrum small-molecule inhibitor of RTK which inhibited vascular endothelial growth factor receptor (VEGFR) [8–10]. Similarly, patients with locoregional clear-cell renal-cell carcinoma at high risk for tumor recurrence after nephrectomy, the median duration of disease-free survival was significantly longer in the sunitinib group than in the placebo group [11]. Unfortunately, the vast majority of treated patients with ccRCC eventually develop progressive disease because of intrinsic resistance or acquired resistance [12]. Hence, there is an urgent need to develop more effective prognostic molecular biomarkers to help identify clinic patients and chemoresistance of ccRCC.

The tumor-node-metastasis (TNM) classification facilitates the identification of patients who is at high risk for recurrence or progression and stratifies the intensity of postoperative surveillance protocols which viewed as a strong, reliable predictor of oncological outcome after surgical extirpation for ccRCC patients [4, 13]. However, the clinical outcomes of patients with ccRCC may vary considerably even within the same tumor stage. Moreover, the TNM cancer staging systems predict survival only bases on anatomic and histological extent of the tumor without molecular changes [9]. Mutation or dysregulation of different gene or protein expression in the same TNM stage may contribute to this diversity of clinical behavior. All of these suggest that further clues other than or combining with TNM staging system is needed for more accurate assessment of prognosis. The stage, size, grade, and necrosis (SSIGN) score was a good model to predict cancer specific survival (CSS) for ccRCC [14, 15], and a new preoperative serum C-reactive protein and the TNM classification (TNM-C Score) was a useful and easy method for predicting outcome in ccRCC [16]. Specific molecular gene markers could improve accuracy of outcome prediction [17, 18]. Therefore, new prognostic molecular markers which could stratify patients precisely are clearly needed.

Neighbor of Brca1 gene (NBR1) has been of interest due to its position close to BRCA1, but has no involvement in breast or ovarian cancer [19]. Recent studies showed that NBR1 was an autophagy receptor for selective autophagosomal degradation of ubiquitinated targets as containing light chain 3 (LC3) and ubiquitin (Ub)-binding domains [20, 21]. It also had a role in regulate growth-factor receptor and downstream signaling pathways in osteoblast differentiation as a scaffold protein [22].

In the present study, NBR1 was downregulated in ccRCC tissues, renal cancer cell lines and their chemoresistance cells. Furthermore, we analyzed the prognostic significance of NBR1 by using independent ways and multiple approaches.

## RESULTS

### NBR1 was downregulated and associated with various clinicopathological parameters in ccRCC

**Figure 1:**
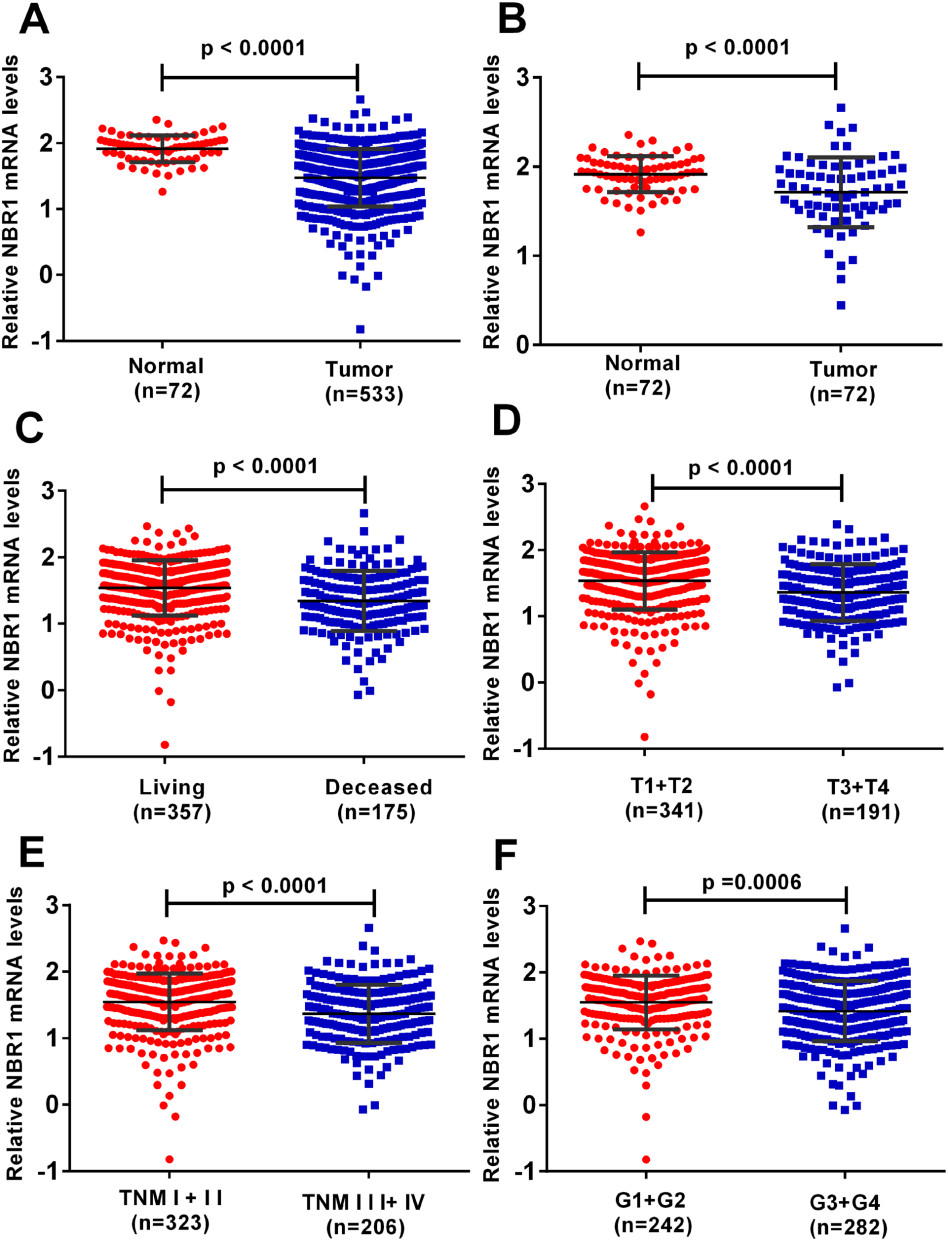
The level of NBR1 is downregulated and correlated with various clinicopathological parameters in ccRCC tissues **(A-F)** The mRNA level of NBR1 in ccRCC was downloaded from the TCGA-KIRC dataset containing 72 normal tissues and 533 ccRCC tissues. The mRNA levels of NBR1 were compared in different clinicopathological parameters: (A) cancer versus para-cancer, (B) cancer versus paired para-cancer, (C) living status, (D) T stage, (E) TNM stage, (F) G stage, data differences were tested with Student’s T-test.

NBR1 mRNA expression in ccRCC cancer tissues and normal tissues was assessed from TCGA-KIRC database which contained 533 cases including 72 paired cases. NBR1 expression was lower in ccRCC tissues compared with normal tissues and in paired ccRCC tissues (Figure 1A, 1B). Next, we analyzed the relationship between clinicopathological parameters and NBR1 expression in ccRCC (Table 1). The database revealed NBR1 expression was significantly lower in deceased compared with living ccRCC patients (Figure 1C). Downregulated NBR1 expression also correlated significantly with higher T stage, pathological TNM stage, and Grade stage in ccRCC (Figure 1D–1F). However, the expression of NBR1 was not associated with gender, age or lymph node metastasis (Supplementary Figure 1). These data indicate that NBR1 expression was downregulated and associated with various clinicopathological parameters in ccRCC.

**Table 1:** Correlation between NBR1 mRNA expression and clinicopathological parameters of ccRCC patients

Parameter		NBR1 mRNA expression			χ^2^	p value
		Number	Low (n=259)	High (n=258)		
Age(years)	<=60	257	127	130		
	>60	260	132	128	0.095	0.792
gender	male	336	170	166		
	female	181	89	92	0.095	0.783
T stage	T1+T2	332	138	194		
	T3+T4	185	121	64	27.006	0.000^*^
N stage	N0+ NX	503	249	254		
	N1	14	10	4	2.619	0.174
M stage	M0+ MX	441	209	232		
	M1	76	50	26	8.777	0.004^*^
G stage	G1+G2	239	99	140		
	G3+G4	278	160	118	13.377	0.000^*^
TNM stage	I+II	314	133	181		
	III+IV	203	126	77	29.793	0.000^*^

### Low level of NBR1 mRNA predicted poor prognosis of ccRCC patients

**Figure 2:**
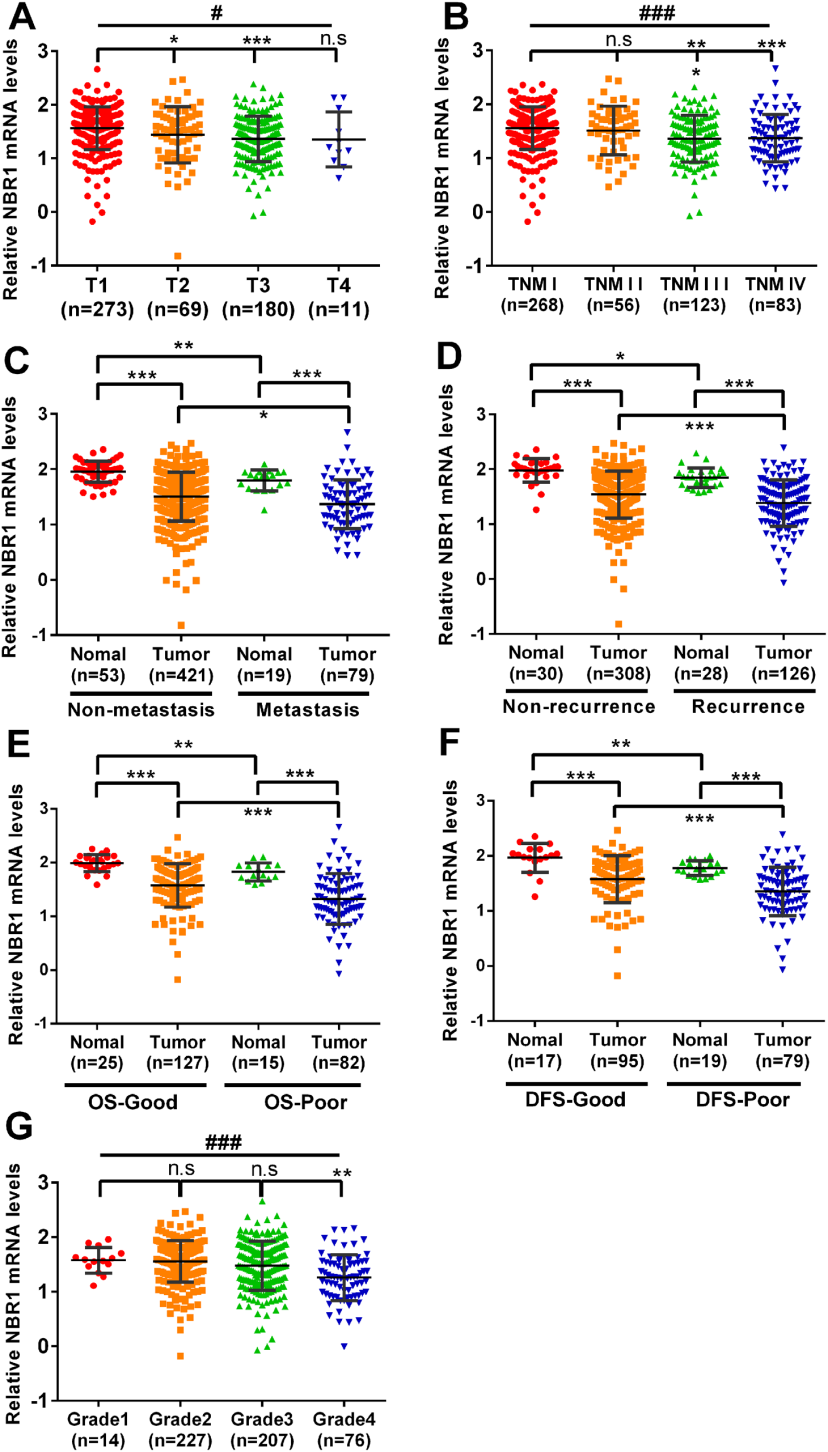
Low level of NBR1 mRNA predicts poor prognosis of ccRCC patients The NBR1 mRNA level was decreased and negatively correlated with TNM stage (one-way ANOVA, p<0.0001, student’s T-test, p<0.05, **(A, B)**. **(C)** Non-metastasis and metastasis, **(D)** non-recurrent and recurrent, **(E)** OS-good and os-poor, **(F)** DFS-good and DFS-poor, **(G)** Grade, data differences were tested with one-way ANOVA or Student’s T-test.

The NBR1 mRNA level was decreased and negatively correlated with the size and weight of tumors (one-way ANOVA, p < 0.001, Figure 2A, 2B). It also exhibited difference between the cancer tissues and normal tissues with non-metastasis and metastasis (Figure 2C). To investigate the prognostic significance of NBR1, we compared NBR1 mRNA level between patients with or without recurrence. NBR1 mRNA expression of cancer tissues and normal tissues in non-recurrent ccRCC was significantly higher in recurrent ccRCC (Figure 2D). Moreover, the mRNA level of NBR1 was able to classify ccRCC patients with a good or poor prognosis both in cancer tissues and normal tissues (Figure 2E, 2F). Similarly, the NBR1 mRNA level was decreased and negatively correlated with the grade of tumors (one-way ANOVA, p < 0.001, Figure 2G), indicating that NBR1 can be a potential prognostic biomarker for ccRCC.

### The association between low NBR1 expression and diagnostic value in ccRCC patients

**Figure 3:**
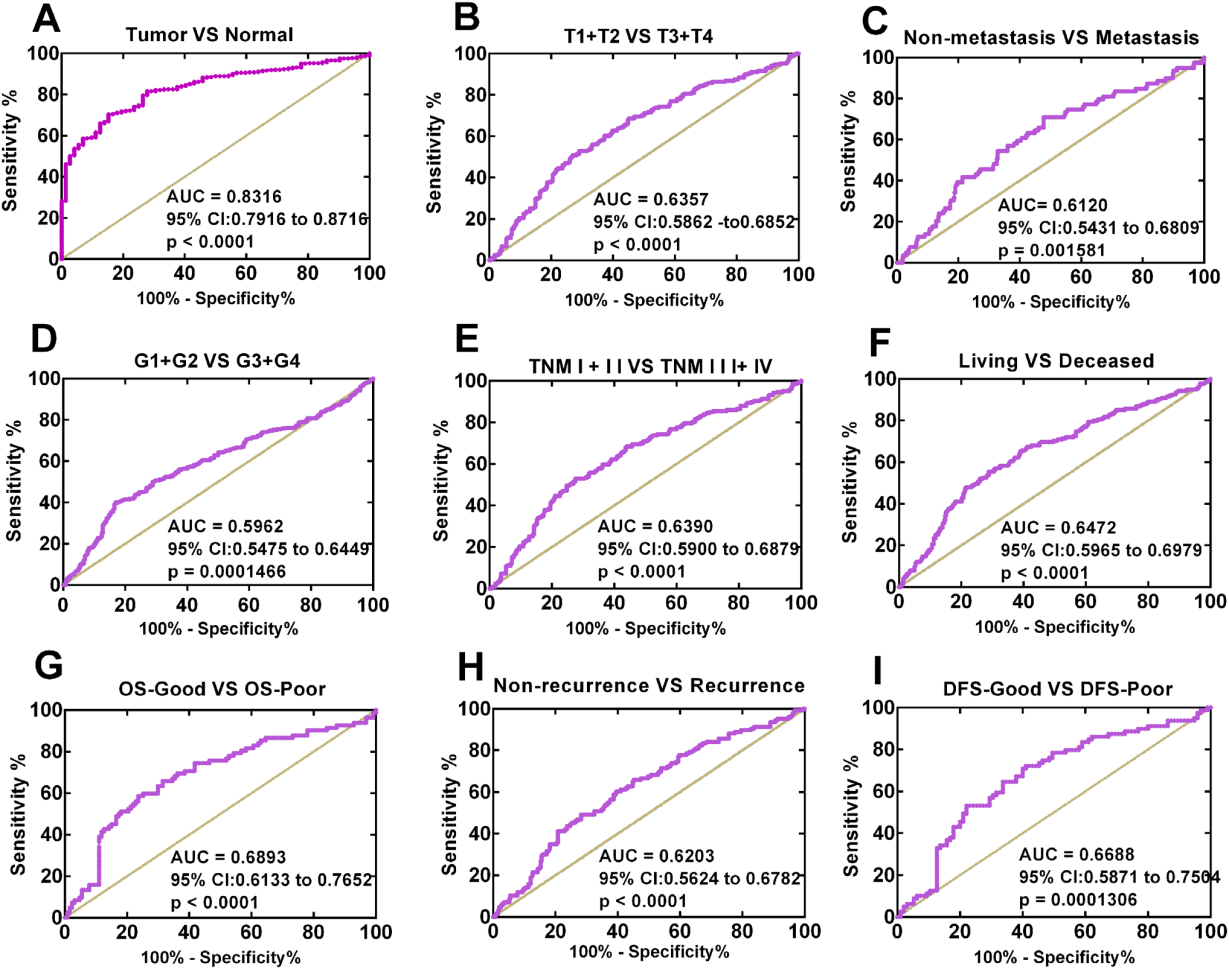
Low NBR1 expression serves as a diagnostic indicator in ccRCC patients **(A)** ROC curve showed that BNR1 could effectively distinguish ccRCC from para-cancer tissues. The AUC was 0.8316 (p < 0.0001). ROC curve analysis towards the expression of NBR1 mRNA in subgroups of ccRCC patients against **(B)** T stage, **(C)** metastasis status, **(D)** G stage, **(E)** TNM stage, **(F)** OS status, **(G)** OS good VS poor, **(H)** recurrence atatus, **(I)** DFS good VS poor.

To explore the diagnostic value of NBR1 in ccRCC, we analyzed the ROC curves for the clinicopathological parameters. NBR1 could effectively differentiate ccRCC from normal tissues yielding an area under the curve (AUC) of 0.8316 (95% CI: 0.7916 to 0.8716; p < 0.0001) with a sensitivity of 70.36% and a specificity of 84.72% when the cutoff value was 1.7223 (Figure 3A). We also analyzed the expression of NBR1 mRNA in subgroups of ccRCC patients against T stage, N stage, TNM stage, metastasis status, G stage, OS status, OS-good or poor prognosis, DFS status, DFS-good or poor prognosis. Those results indicated that low NBR1 expression could be a potential diagnostic indicators for ccRCC patients with (T1 + T2) / (T3+T4) stage (Figure 3B, AUC= 0.6357, p < 0.0001), TNM (I+II) / (III+IV) stage (Figure 3C, AUC= 0.6390, p < 0.0001), non-metastasis / metastasis (Figure 3D, AUC= 0.6120, p = 0.001581), (G1+G2) / (G3+G4) stage (Figure 3E, AUC= 0.5962, p =0.0001466), OS living / deceased status (Figure 3F, AUC= 0.6472, p < 0.0001), OS-good / poor prognosis (Figure 3G, AUC= 0.6893, p < 0.0001), DFS status (Figure 3H, AUC=0.6203, p < 0.0001), DFS-good / poor prognosis (Figure 3I, AUC=0.6688, p = 0.0001306). However, low NBR1 level could not differentiate ccRCC patients with N1 stage from ccRCC patients with N0 stage (data not shown).

### The correlation between low NBR1 expression and poor overall survival in ccRCC patients

**Figure 4:**
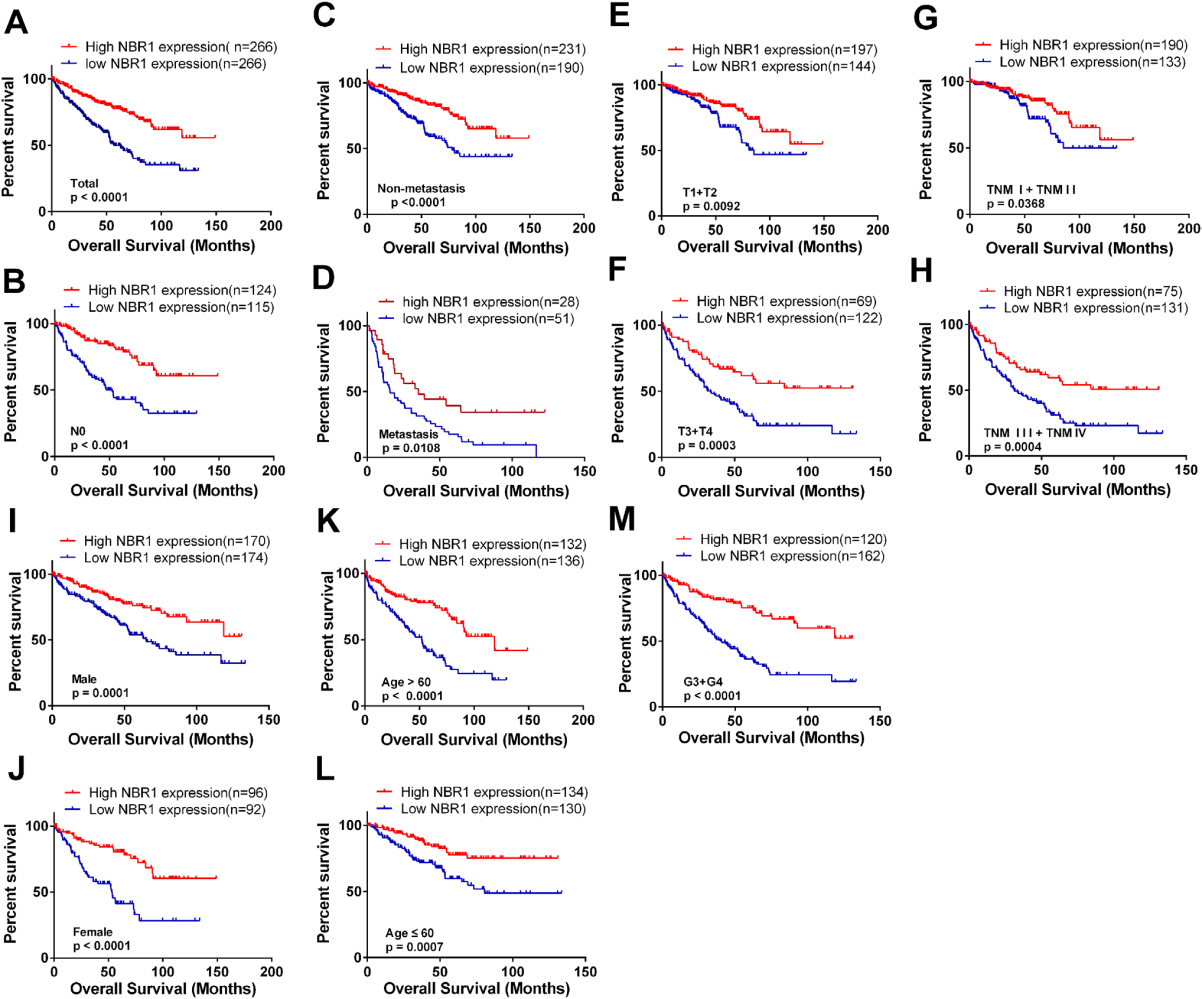
Low level of NBR1 mRNA predicts poor overall survival rate in ccRCC patients **(A)** The ccRCC patients from TCGA-KIRC database were divided into low NBR1 expression group and high NBR1 expression group according to the median expression value of NBR1 mRNA level. The correlation between NBR1 expression and overall survival time of total ccRCC patients was analyzed by Kaplan-Meier. **(B-M)** overall survival analysis towards the expression of NBR1 mRNA was performed in subgroups of ccRCC patients: (B) N0 stage, (C) non-metastasis status, (D) metastasis status, (E) T1+T2 stage, (F) T3+T4 stage, (G) TNM (I+II), (H) TNM (III+IV), (I) Male, (J) Female, (K) Age > 60 years, (L) Age ≤ 60 years, (M) G3+G4 stage.

KM survival curves were plotted to evaluate the association of NBR1 mRNA expression level with the overall survival time of ccRCC patients. Total 533 ccRCC patients from TCGA-KIRC database were divided into ‘high’ and ‘low’ groups based on the median values of NBR1 mRNA. Patients with low NBR1 mRNA level had shorter OS time (Figure 4A, log-rank test, p < 0.0001). Moreover, we conducted overall survival analysis towards the expression of NBR1 mRNA in subgroups of ccRCC patients. Our results showed that low NBR1 expression could be a potential prognostic factor for ccRCC patients with N0 stage (Figure 4B, p < 0.0001), non-metastasis (Figure 4C, p < 0.0001), metastasis (Figure 4D, p = 0.0108), T1 + T2 stage (Figure 4E, p = 0.0092), T3 + T4 stage (Figure 4F, p = 0.0003), TNM (I+II) (Figure 4G, p = 0.0368), TNM (III+IV) stage (Figure 2H, p = 0.0004), Male (Figure 4I, p = 0.0001), female (Figure 4J, p < 0.0001), Age > 60 years (Figure 4K, p < 0.0001), Age ≤ 60 years (Figure 4L, p = 0.0007), G3+G4 stage (Figure 4M, p < 0.0001). However, low NBR1 expression had no significant correlation with OS of ccRCC patients with N1 stage, G1+G2 stage, (Supplementary Figure 2A, 2B, 2C).

### The correlation between low NBR1 expression and poor disease-free survival in ccRCC patients

**Figure 5:**
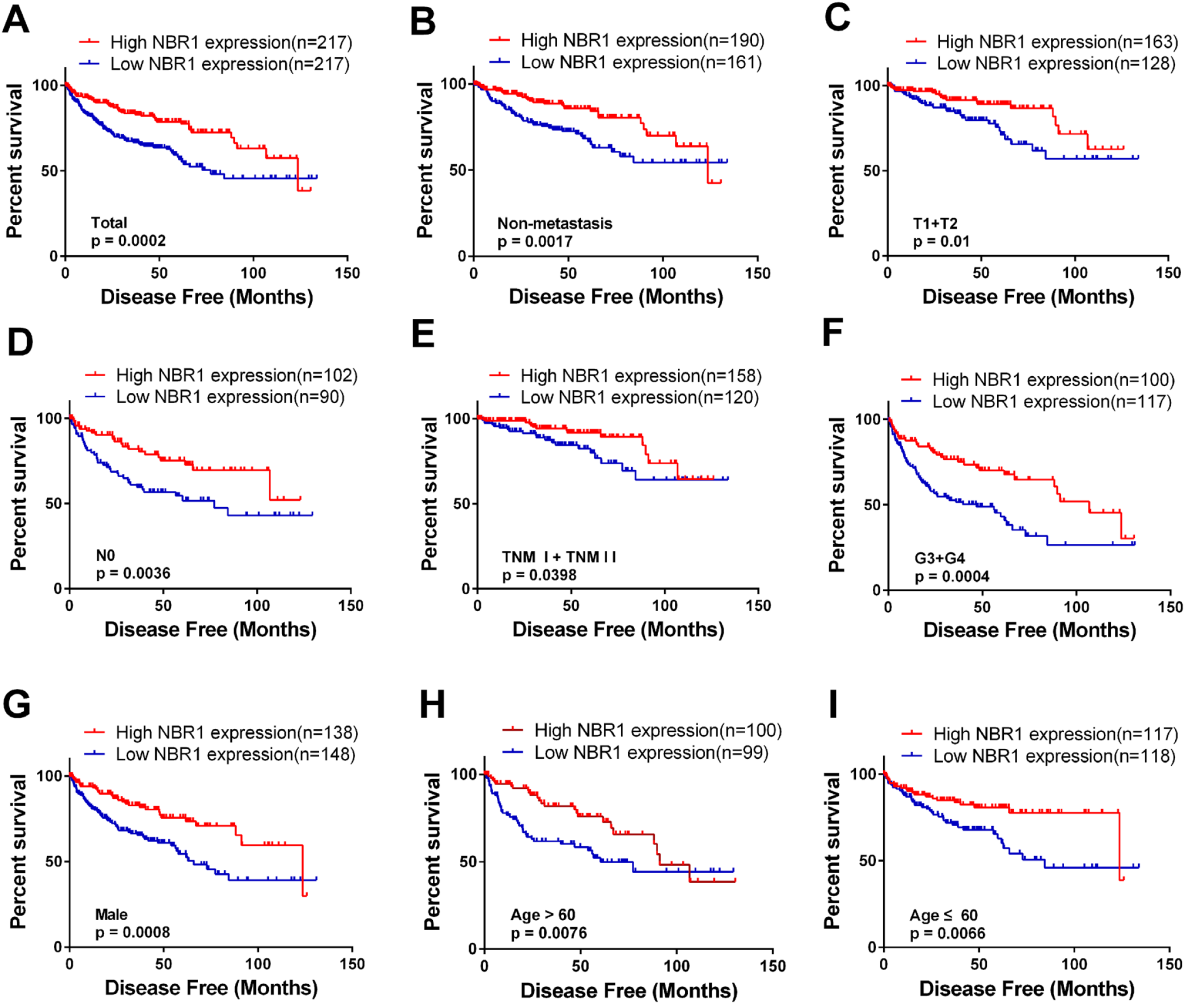
Low level of NBR1 mRNA predicts poor disease-free survival rate in ccRCC patients **(A)** The ccRCC patients from TCGA-KIRC database were divided into low NBR1 expression group and high NBR1 expression group according to the median expression value of NBR1 mRNA level. The correlation between NBR1 expression and disease-free survival time of total ccRCC patients was analyzed by Kaplan-Meier. **(B-I)** disease-free survival analysis towards the expression of NBR1 mRNA was performed in subgroups of ccRCC patients: (B) non-metastasis status, (C) T1+T2 stage,, (D) N0 stage, (E) TNM (I+II), (F) G3+G4 stage, (G) Male, (H) Age > 60 years, (I) Age ≤ 60 years.

To test the prognostic value of NBR1, the correlation between NBR1 expression and disease-free survival (DFS) time of ccRCC patients was analyzed with Kaplan-Meier. According to the median expression value of NBR1 mRNA level, the total 434 ccRCC patients from TCGA-KIRC database were divided into ‘high’ and ‘low’ NBR1 expression group. Low NBR1 expression group had poorer DFS than high NBR1 expression group (Figure 5A, log-rank test, P < 0.0001). Moreover, we conducted DFS analysis in subgroups of ccRCC patients and low NBR1 expression could be a potential prognostic factor for ccRCC patients with non-metastasis (Figure 5B, p = 0.0017), T1 + T2 stage (Figure 5C, p = 0.01), N0 stage (Figure 5D, p = 0.0036), TNM (I+II) (Figure 5E, p = 0.0398), G3+G4 stage (Figure 5F, p = 0.0004), Male (Figure 5G, p = 0.0008), Age > 60 years (Figure 5H, p = 0.0076), Age ≤ 60 years (Figure 5I, p = 0.0066). However, low NBR1 expression had no significant correlation with DFS of ccRCC patients with N1 stage, metastasis, G1+G2 stage, T3+T4 stage, TNM (III+IV) or female (Supplementary Figure 3A-3E).

### NBR1 was an independent prognostic marker for ccRCC

The association between NBR1 mRNA and OS (Table 2) or DFS (Table 3) in the cohort of ccRCC patients was investigated with univariate and multivariate analyses. Total ccRCC patients from TCGA-KIRC database were divided into ‘high’ and ‘low’ NBR1 expression group according to the median value. Univariate analysis indicated that the patients with low NBR1 level exhibited a shorter OS and DFS [NBR1 mRNA OS: hazard ratio (HR) 0.396, p = 0.000 and DFS HR 0.477, p = 0.000]. When controlling for other variables in the multivariate analysis, low NBR1 mRNA level retained its clinical significance as a marker of shorter survival (mRNA OS: HR 0.483, p = 0.000 and DFS HR 0.641, p = 0.022). Taken together, these data suggested that low NBR1 expression level is an independent predictor of poor prognosis for ccRCC patients

**Table 2:** Univariate and multivariate analyses of NBR1 mRNA level and patient overall survival

Variable	Univariate analysis			Multivariate analysis^c^		
	HR^a^	95%CI^b^	P	HR	95% CI	P
Overall survival (n = 517)						
Age (years)						
≤60 (n = 257)	0.586	0.416-0.771	0.000^*^	0.608	0.445-0.830	0.002^*^
>60 (n = 260)						
Gender						
Female (n = 181)	0.965	0.707-1.318	0.825			
Male (n = 336)						
T stage						
T1 or T2 (n = 332)	0.329	0.242-0.445	0.000^*^	0.682	0.447-0.976	0.037^*^
T3 or T4 (n = 185)						
N stage						
N0 or NX (n = 503)	0.281	0.148-0.534	0.000^*^			
N1 (n = 14)						
M stage						
M0 or MX (n = 441)	0.229	0.167-0.313	0.000^*^	0.346	0.243-0.493	0.000^*^
M1 (n = 76)						
G grade						
G1 or G2 (n = 239)	0.384	0.273-0.540	0.000^*^	0.576	0.402-0.825	0.003^*^
G3 or G4 (n = 278)						
NBR1						
High(n = 259)	0.396	0.287-0.547	0.000^*^	0.483	0.348-0.672	0.000^*^
Low (n = 258)						

Univariate and multivariate analyses of NBR1 mRNA level and patient overall survival.

^a^ Hazard ratio, estimated from Cox proportional hazard regression model.

^b^ Confidence interval of the estimated HR.

^c^ Multivariate models were adjusted for T, N, M classification, age and gender.

**Table 3:** Univariate and multivariate analyses of NBR1 mRNA level and patient disease–free survival

Variable	Univariate analysis			Multivariate analysis^c^		
	HR^a^	95%CI^b^	P	HR	95% CI	P
Disease–free survival (n = 421)						
Age (years)						
≤60 (n = 228)	0.734	0.515-1.045	0.086			
>60 (n = 193)						
Gender						
Female (n = 142)	1.421	0.956-2.111	0.082			
Male (n = 279)						
T stage						
T1 or T2 (n = 282)	0.222	0.154-0.321	0.000^*^	0.511	0.335-0.780	0.002^*^
T3 or T4 (n = 139)						
N stage						
N0 or NX (n = 409)	0.169	0.085-0.337	0.000^*^	0.303	0.145-0.631	0.001^*^
N1 (n = 12)						
M stage						
M0 or MX (n = 370)	0.118	0.081-0.171	0.000^*^	0.203	0.133-0.308	0.000^*^
M1 (n = 51)						
G grade						
G1 or G2 (n = 207)	0.298	0.198-0.450	0.000^*^	0.42	0.274-0.643	0.000^*^
G3 or G4 (n = 214)						
NBR1						
High (n = 211)	0.477	0.330-0.690	0.000^*^	0.641	0. 438-0.938	0.022^*^
Low (n = 210)						

Univariate andmultivariate analyses of NBR1 mRNA level and patient disease–free survival.

^a^ Hazard ratio, estimated from Cox proportional hazard regression model.

^b^ Confidence interval of the estimated HR.

^c^ Multivariate models were adjusted for T, N, M classification, age and gender.

### Low level of NBR1 expression was validated in ccRCC cells and tissues

**Figure 6:**
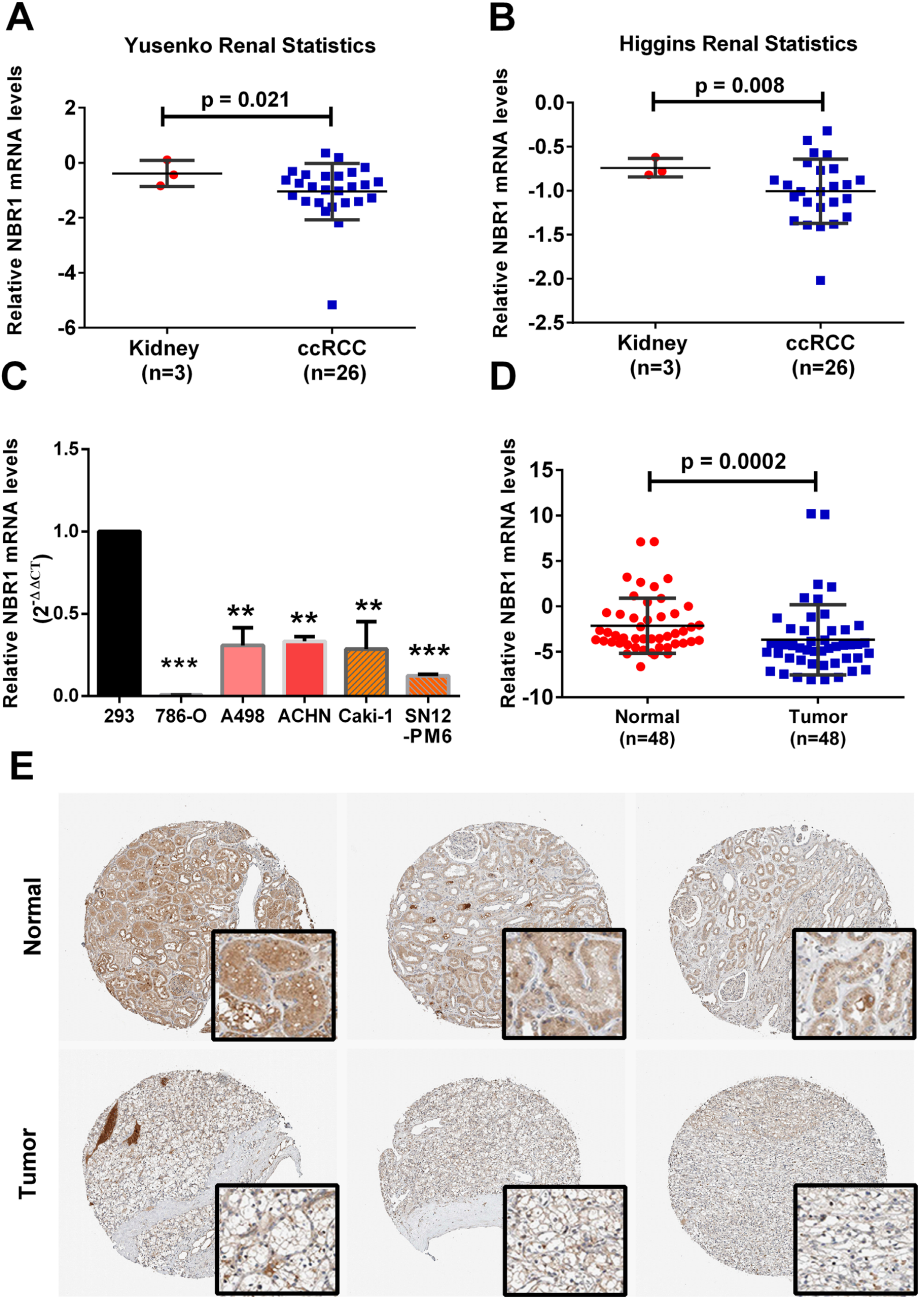
NBR1 is downregulated in ccRCC cells and tissues **(A, B)** Gene expression levels of NBR1 in up to two additional ccRCC data sets. **(C)** Gene expression levels of NBR1 in renal cancer cell lines. **(D)** Gene expression levels of NBR1 in ccRCC tissues. **(E)** Immunohistochemistry (IHC) analysis of NBR1 expression in ccRCC tissues and para-cancer tissues. Representative images were shown. Data differences were tested with Student’s T-test (^***^, p<0.001, ^**^, p<0.01, ^*^, p<0.05).

To further confirm the results of the TCGA-KIRC database, we next extended this observation to two additional data sets in oncomine datebase (https://www.oncomine.org). NBR1 was lower in the ccRCC tissues of the two data sets (Figure 6A, 6B). Then we tested NBR1 mRNA in ccRCC cells and ccRCC tissues in our own patient samples (Figure 6C, 6D). IHC assay of ccRCC tissues and normal tissues was assessed from The Human Protein Atlas (http://www.proteinatlas.org) (Figure 6E). All those results revealed that NBR1 levels of ccRCC cells and tissues were significantly lower than immortalized renal epithelial cells and normal tissues.

### NBR1 was downregulated in sunitinib-resistant cell lines

**Figure 7:**
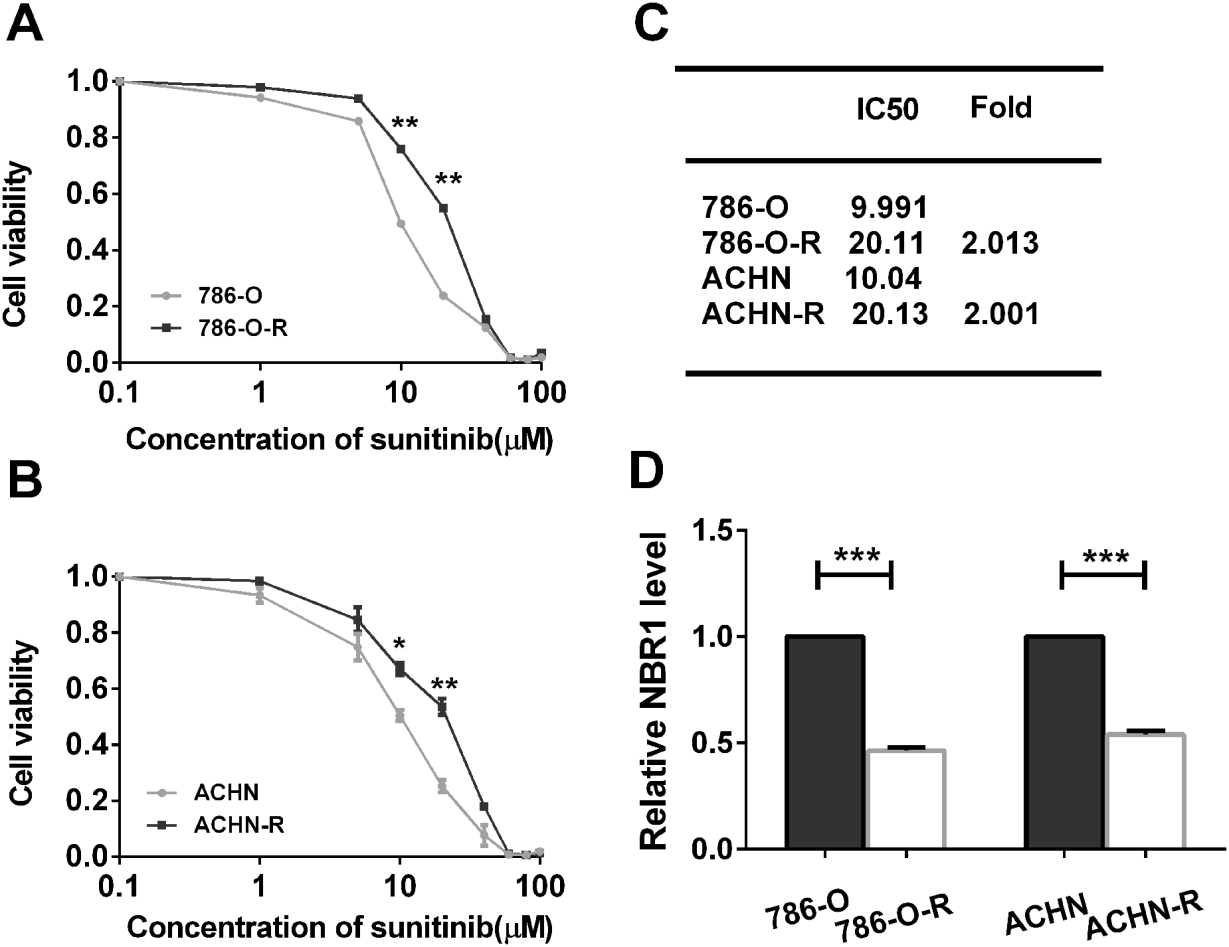
The sunitinib sensitivity curve and NBR1 expression level of sunitinib-resistant and parental cells and correlated with the VEGF signaling pathway **(A)** The sunitinib sensitivity curve of 786-O and 786O-R. **(B)** The sunitinib sensitivity curve of ACHN and ACHN-R. **(C)** IC50 value of sunitinib-resistant and parental cells. The error bars represent mean±SD of three independent experiments. **(D)** Gene expression of NBR1 in sunitinib-resistant and parental cells. Data differences were tested with Student’s T-test (^***^, p<0.001, ^**^, p<0.01, ^*^, p<0.05).

Two sunitinib-resistant cell lines (786-O and ACHN) were established by continuous low-dose stimulation in conjunction with intermittent high-dose pulses of sunitinib as described and characterized who are designated as 786-O-R and ACHN-R cells, respectively [23]. Then, we evaluated sunitinib sensitivity of sunitinib-resistant cells and the parental cells. 786-O-R and ACHN-R showed higher cell viability compared with the parental cells after various concentrations of sunitinib administration (Figure 7A, 7B). 786-O-R and ACHN-R had a more than 2-fold IC50 values to the parental cells (Figure 7C). NBR1 mRNA expression in sunitinib-resistant cells exhibited a significant lower of that in the corresponding parental cells by qRT-PCR (Figure 7D). These results showed that sunitinib-resistant cell lines were successfully constructed, sunitinib-resistant cells had higher sunitinib tolerance and had a lower NBR1 expression compared with corresponding parental cell lines.

## DISCUSSION

NBR1 is originally cloned as a candidate gene for the ovarian cancer antigen and its position close to BRCA1, two isoforms of NBR1A and NBR1B are downregulated in malignant mammary tissues when compaired with normal cells [19, 24]. And there is a positive correlation between NBR1 and BRCA1 expression in clear cell renal cell carcinoma in supplementary (R = 0.355, p < 0.0001, Supplementary Figure 4). NBR1 is a multidomain protein which had several putative protein–protein interaction modules, such as an N-terminal phox/Bem1p (PB1) domain, a ZZ-type zinc finger (ZZ), a coiled-coiled (CC) region, autophagy receptor containing LC3-binding domain and C-terminal ubiquitin-associated (UBA) domain [20, 22, 25], however, the function of NBR1 in renal cancer remains unknown.

RTKs are major superfamily of membrane-spanning growth factor receptors, which regulate cellular processes such as differentiation, proliferation, migration, and survival. RTKs include insulin-like growth factor receptor (IGFR), epidermal growth factor (EGF) receptor (EGFR), fibroblast growth factor (FGF) receptor (FGFR), platelet-derived growth factor receptor (PDGFR), and vascular endothelial growth factor receptor (VEGFR) [26]. And sunitinib is used as a first line tyrosine kinase inhibitor of VEGFR in renal cancer. NBR1 is a specific late endosomal protein involved in RTK degradation in autophagy by interacting and colocalizing with spred2 at critical C terminus of EVH1 domain establishing it as a general RTK antagonist [25], but inhibition of RTK degradation on C-terminal 133 amino acids of NBR1 [27]. Spred2 is shown to inhibit ERK1/2 downstreamof only a subset of RTK growth factors such as FGF and VEGF [28].

ccRCC is a malignant kidney cancer distinguished by early loss of Von Hippel-Lindau (VHL) tumor suppressor protein, leading to accumulation of the hypoxia-inducible transcription factor (HIF) [29–31], ccRCC is composed of lipid droplets in cell cytoplasm and HIF2α promoted lipid storage [32]. Autophagy is particularly active during metabolic stress which cells capture intracellular proteins, lipids and organelles, and deliver them to the lysosomal compartment where they are degraded [33]. In the cancer cell, it fulfils a dual role in tumor-promoting and tumor-suppressing. Autophagy can mediate HIF2α degradation [34] and rapidly clear lipid droplets to suppress clear cell renal cell carcinomas and promote patient survival [35].

P62 is a multifunctional protein which participates in autophagy and signal transduction [36] as its levels are elevated in almost all human tumors tested so far and required for tumor growth and metastases [37, 38]. Multicenter I/IIa trial had already evaluated safety and clinical activity of Elenagen who had been employed a plasmid DNA vaccine as a platform for p62 expression in patients with advanced solid tumors including breast, ovary, lung, melanoma and renal cancer [38]. NBR1 and p62 interact with LC3 and bind to ubiquitin-marked autophagic substrates via C-terminal ubiquitin-associated (UBA) domain and deliver them to autophagosomes for degradation [20, 22]. NBR1 differs from p62 in its UBA structure and with a much higher affinity for ubiquitin, which suggests that NBR1 may form intracellular inclusions with ubiquitylated autophagic substrates more efficiently than p62 [39]. Full-length NBR1 complexed with activated p-p38 MAPK while homologous recombination truncated (trNbr1) lacking LIR and UBA domain but containing an intact PB1 domain can still bind p62 and enhanced p38 MAPK activity to increase cell differentiation [22], we found that the mRNA of p62 had a negative correlation with that of NBR1 in TCGA datebase (R = -0.168, p < 0.0001, Supplementary Figure 5). NBR1 is recognized as a regulator of diverse cellular kinase signaling pathways with multiply domains now and its role in cancer need more attention.

In this study, NBR1 was downregulated in ccRCC tissues, renal cancer cell lines and their sunitinib-resistant cells. The low NBR1 level was strongly associated with a poor clinical outcome of ccRCC patients. NBR1 could discriminate metastasis, recurrence, and prognosis in ccRCC patients and chemoresistance in renal cells. The detail role in ccRCC remains unknown.

The drawback of this study was the lack of enough specimens for NBR1 protein level and OS, DFS time of KM-curve in protein level. Clarify the role of NBR1 in ccRCC tumorigenesis and metastasis, chemoresistance need to be further investigated.

In conclusion, our results provide the idea that NBR1 is downregulated in ccRCC tissues, renal cancer cell lines and their sunitinib-resistant cells and identified as an new independent predictor for prognosis in ccRCC patients. These findings will facilitate patient counseling and individualize the management of patients with ccRCC.

## MATERIALS AND METHODS

### Patient samples

Surgical specimens (48 paired human ccRCC tissues and adjacent normal tissues) were obtained from 2010-2016 in Department of Urology, Union Hospital, Tongji Medical College (Wuhan, China). Adjacent normal tissues were acquired at least 5cm away from the tumor site and freshly frozen in liquid nitrogen then stored at -80°C for RNA extraction. Informed consent was obtained from patients and the study was approved by the Institutional Review Board of Huazhong University of Science and Technology.

### RNA extraction and qRT-PCR

Total RNA of tissues was extracted with the TRizol reagent (Thermo, Massachusetts, USA) according to the manufacturer’s instructions. The concentration and purity of the RNA solution were measured by the NanoDrop 2000 spectrophotometer (NanoDrop Technologies, Wilmington, USA). 1 μg of enriched tissue or cell RNAs were applied for reverse transcription. qPCR analysis was performed (LightCycler 480II; Roche, Basel, Switzerland) with the SYBR Green mix (Thermo, Massachusetts, USA) according to the manufacturer’s instructions. Relative expression of NBR1 was calculated using the power formula normalized to GAPDH: 2^-ΔCt^ (ΔCt = Ct_NBR1_–Ct_GAPDH_). Gene primers were purchased from GENEWIZ (GENEWIZ, Suzhou, China):

NBR1 Forward 5’-GTGCAGTCGTTTCCACTTGT-3’

 Reverse 5’-GGATGGGTTCTGGAGGACAA-3’

GAPDH Forward 5’-GAGTCAACGGATTTGGTCGT-3’

 Reverse 5’-GACAAGCTTCCCGTTCTCAG-3’

### Cell culture and drug intervention

The human renal cancer cell lines 786-O, ACHN, A498, Caki-1, SN12-PM6 and HEK-293 were purchased from The American Type Culture Collection (ATCC, USA). Cells were maintained in DMEM high glucose medium (Google Biotechnology Co., Ltd, Wuhan, China) containing 10% FBS (Lilac Garden Technology Co., Ltd, Wuhan, China) and 1% penicillin-streptomycin at 37°C in a 5% CO2 incubator. For drug intervention, sunitinib (Pfizer) was dissolved in DMSO at a concentration of 15 mg / ml and then added to the culture medium of renal cancer cells at the indicated concentrations.

### Establishment of sunitinib-resistant cell lines

Sunitinib-resistant renal carcinoma cell lines in 786-O and ACHN were established by continuous low-dose stimulation in conjunction with intermittent high-dose pulses of sunitinib as described and characterized [23]. The IC50 values of the sunitinib-resistant cell lines and the parental cell lines are more than twice.

### Bioinformatics analysis

The RNA-seq data of genes in ccRCC patients, normal kidney tissues and clinical information about recurrence, metastasis, overall survival (OS), disease–free survival (DFS) of patients were downloaded from TCGA-KIRC Data Portalt (http://www.cbioportal.org/public-porta).

### Statistical analysis

The data of four groups were analyzed by ANOVA analysis and the data of two groups were analyzed by T-test. The results of IHC and RNA of unpaired samples were analyzed by independent sample t-test or one-way ANOVA. Receiver operator characteristic (ROC) curve and area under the curve (AUC) analyses were applied to detect the optimal cutoff point that yielded the highest total accuracy with respect to discriminating different clinical classifications, good (≥5 years, living) and poor (≤2 years, die) prognosis, respectively. The Kaplan–Meier (KM) curve was generated to evaluate the association between the expression level of NBR1 and the survival rate with log-rank test. Univariate and multivariate Cox proportional hazard regression analyses were used to estimate the prognostic significance of NBR1 in ccRCC. Statistical significance was set at two-tailed, p < 0.05. All statistical analyses were performed using SPSS Statistics 22.0 (IBM SPSS, Chicago, IL).

## SUPPLEMENTARY MATERIAL FIGURES



## Abbreviations

ccRCC: clear cell renal cell carcinoma
HR: hazard ratio
RCC: renal cell carcinoma
NBR1: neighbor of Brca1 gene
TCGA: The Cancer Genome Atlas
VHL: Von Hippel-Lindau
