# Supplementary material for: Low neighbor of Brca1 gene expression predicts poor clinical outcome and resistance of sunitinib in clear cell renal cell carcinoma
Source: Oncotarget. 2017 Oct 23;8(55):94819–33. doi: 10.18632/oncotarget.21999 (PMC5706915; doi:10.18632/oncotarget.21999)
Supplement: Supplementary file 1 [file oncotarget-08-94819-s001.pdf]

Low neighbor of Brca1 gene expression predicts poor clinical outcome and resistance of sunitinib in clear cell renal cell carcinoma

SUPPLEMENTARY MATERIALS

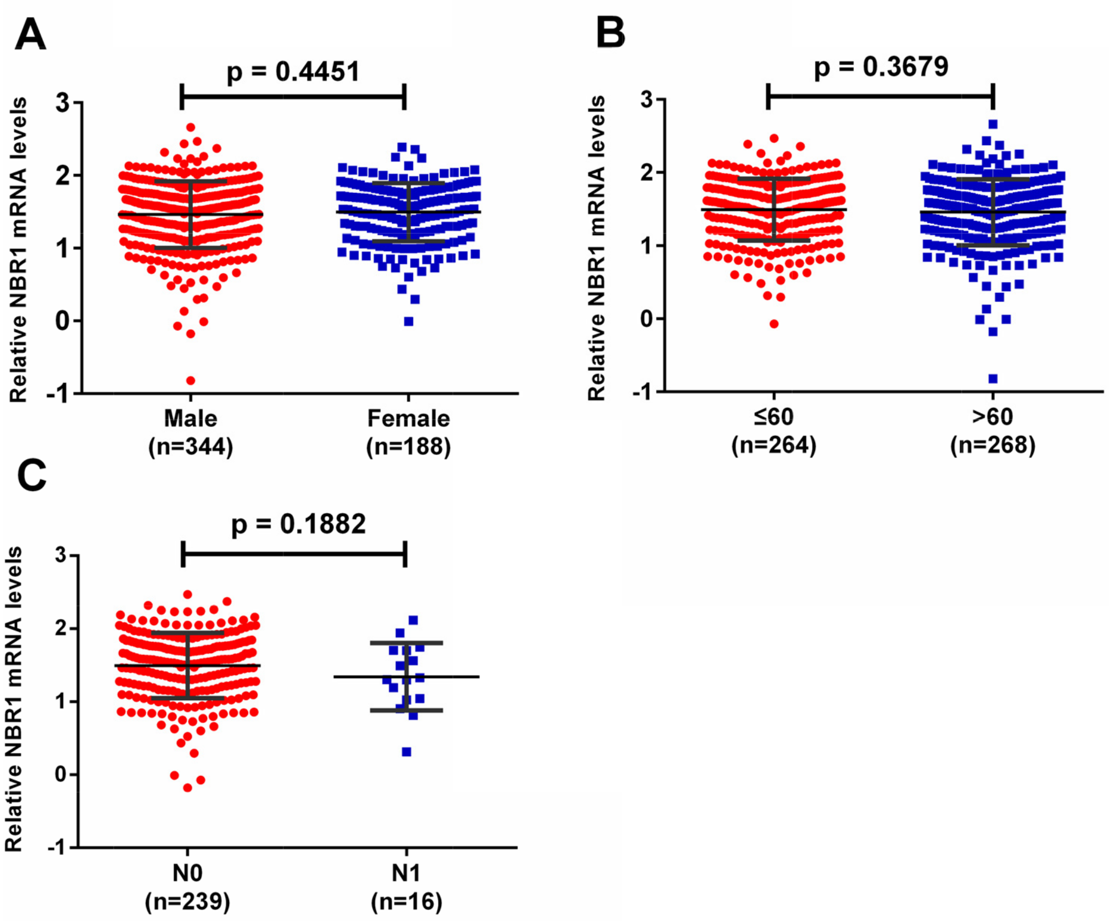

Supplementary Figure 1: The relationship between expression of NBR1 and gender, age, lymph node metastasis.

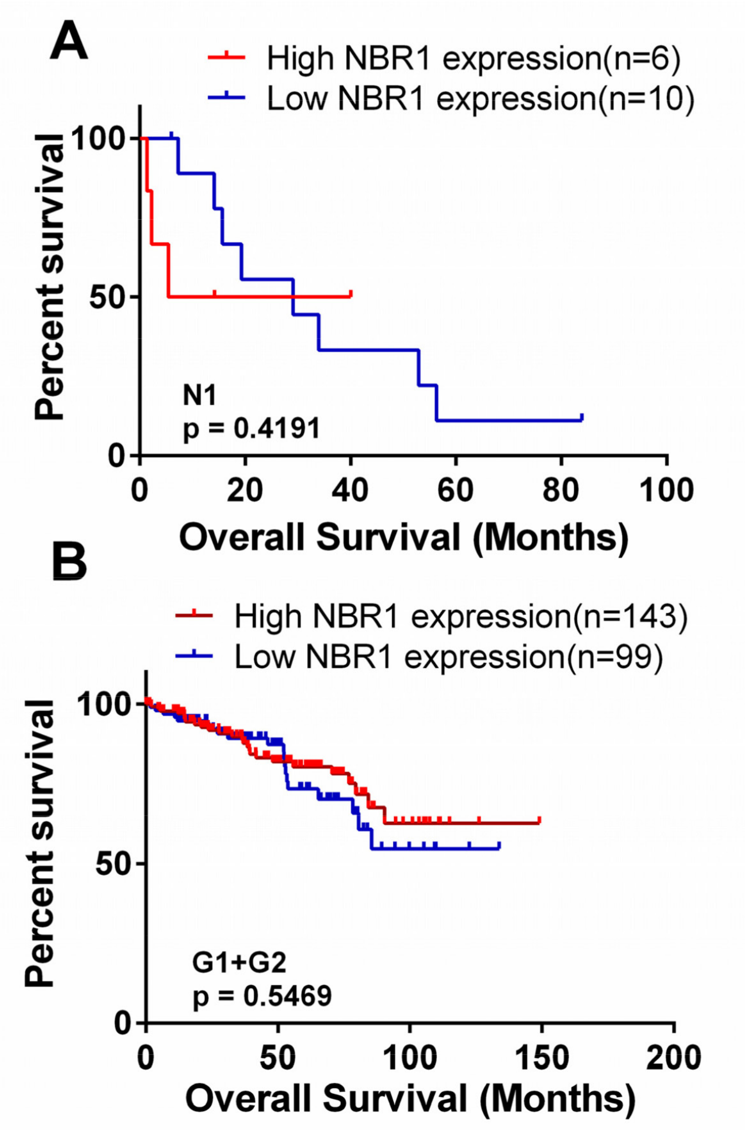

Supplementary Figure 2: The correlation between NBR1 expression and OS of ccRCC patients with N1 stage and G1+G2 stage.

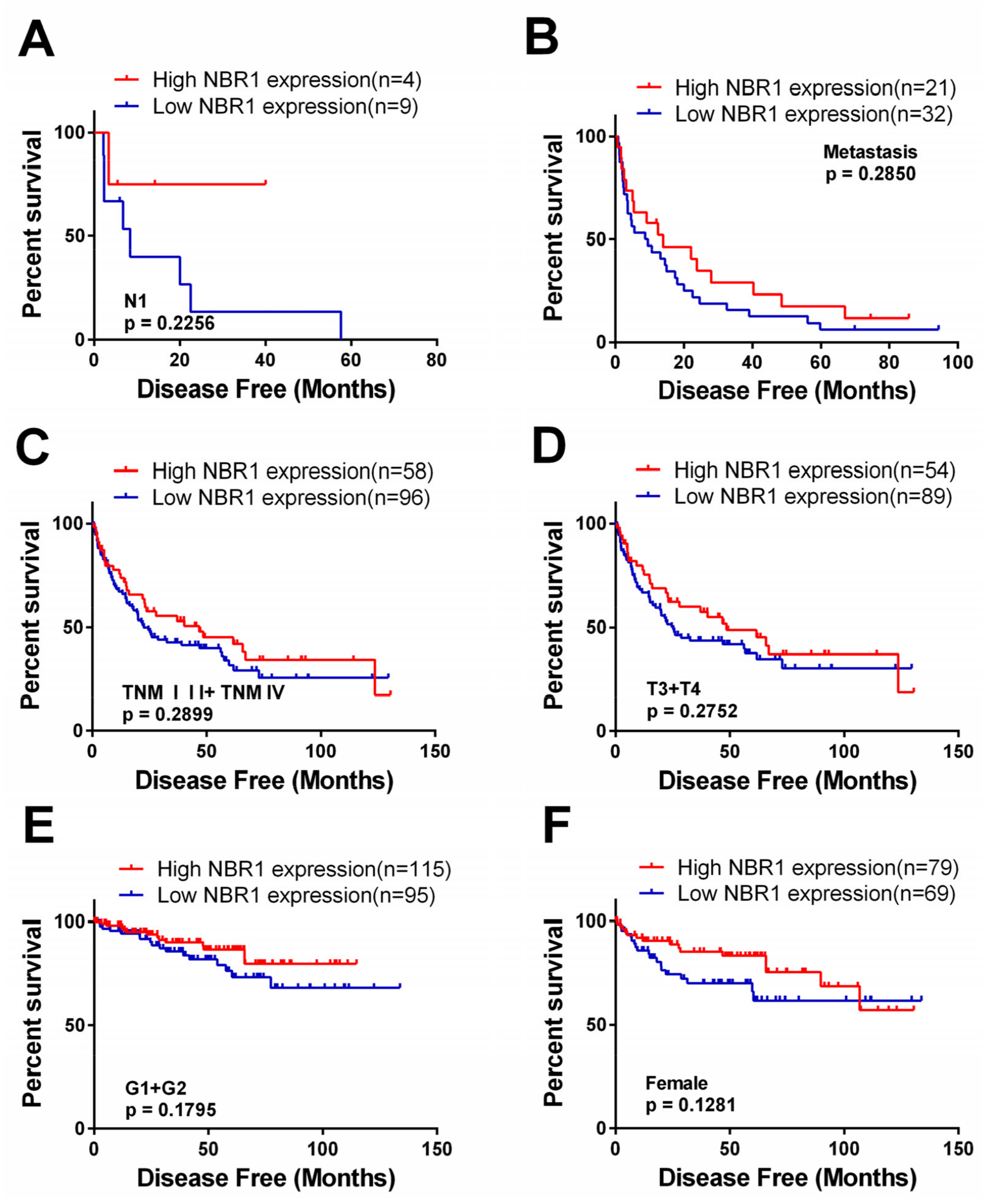

Supplementary Figure 3: The correlation between NBR1 expression and DFS of ccRCC patients with N1 stage, metastasis, TNM (III+IV), T3+T4 stage, G1+G2 stage or female.

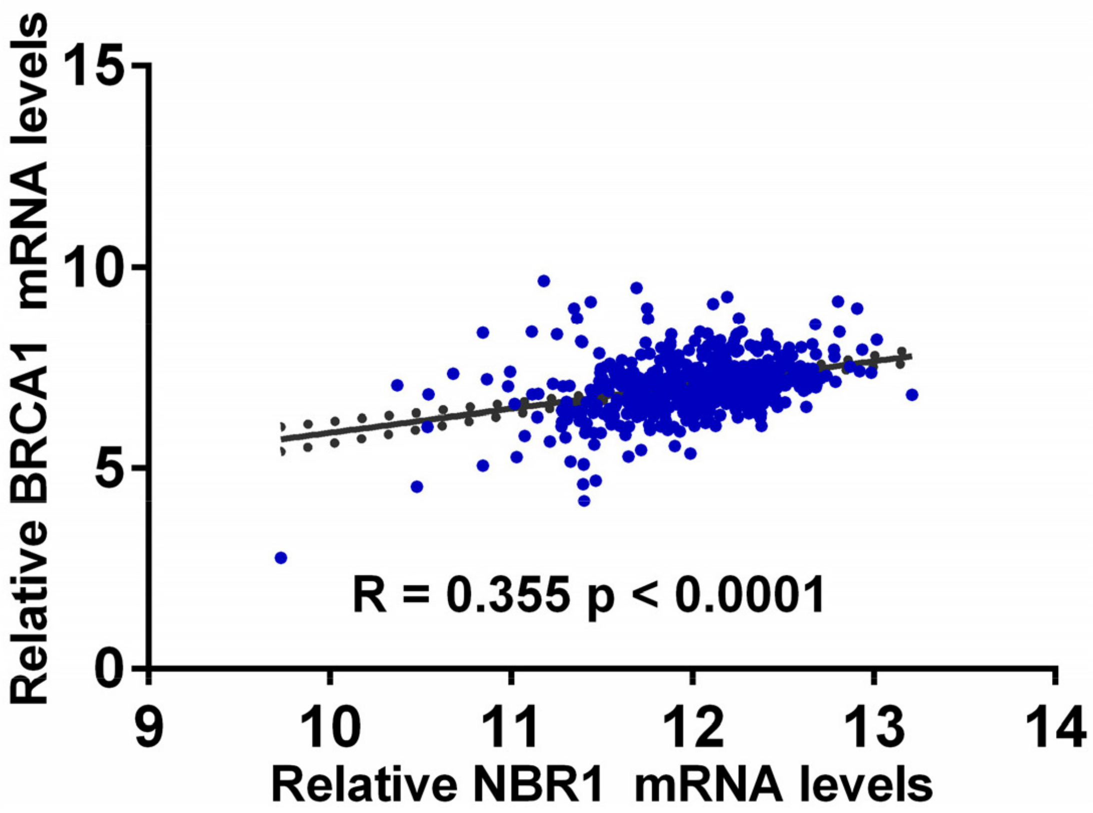

Supplementary Figure 4: The positive correlation between NBR1 and BRCA1 expression in TCGA-KIRC database.

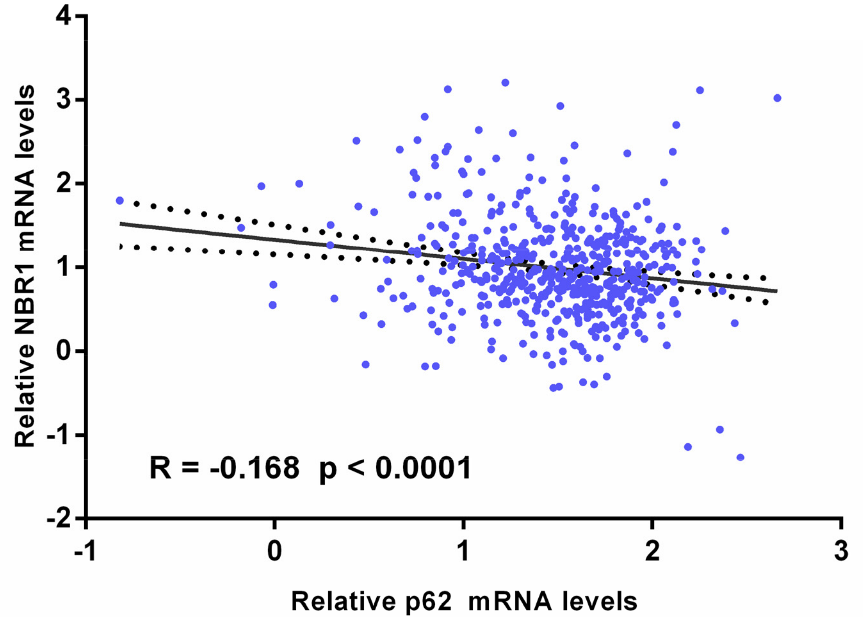

Supplementary Figure 5: The negative correlation between NBR1 and p62 expression in TCGA-KIRC database.
